# Enhancement of dynein-mediated autophagosome trafficking and autophagy maturation by ROS in mouse coronary arterial myocytes

**DOI:** 10.1111/jcmm.12326

**Published:** 2014-06-09

**Authors:** Ming Xu, Xiao-Xue Li, Yang Chen, Ashley L Pitzer, Yang Zhang, Pin-Lan Li

**Affiliations:** Department of Pharmacology and Toxicology, School of Medicine, Virginia Commonwealth UniversityRichmond, VA, USA

**Keywords:** dynein, autophagy maturation, ROS, NAADP, coronary arterial myocyte, high glucose

## Abstract

Dynein-mediated autophagosome (AP) trafficking was recently demonstrated to contribute to the formation of autophagolysosomes (APLs) and autophagic flux process in coronary arterial myocytes (CAMs). However, it remains unknown how the function of dynein as a motor protein for AP trafficking is regulated under physiological and pathological conditions. The present study tested whether the dynein-mediated autophagy maturation is regulated by a redox signalling associated with lysosomal Ca^2+^ release machinery. In primary cultures of CAMs, reactive oxygen species (ROS) including H_2_O_2_ and O_2_^−.^ (generated by xanthine/xanthine oxidase) significantly increased dynein ATPase activity and AP movement, which were accompanied by increased lysosomal fusion with AP and APL formation. Inhibition of dynein activity by (erythro-9-(2-hydroxy-3-nonyl)adenine) (EHNA) or disruption of the dynein complex by dynamitin (DCTN2) overexpression blocked ROS-induced dynein activation, AP movement and APL formation, and resulted in an accumulation of AP along with a failed breakdown of AP. Antagonism of nicotinic acid adenine dinucleotide phosphate (NAADP)-mediated Ca^2+^ signalling with NED-19 and PPADS abolished ROS-enhanced lysosomal Ca^2+^ release and dynein activation in CAMs. In parallel, all these changes were also enhanced by overexpression of NADPH oxidase-1 (Nox1) gene in CAMs. Incubation with high glucose led to a marked O_2_^−.^ production compared with normoglycaemic CAMs, while Nox1 inhibitor ML117 abrogated this effect. Moreover, ML117 and NED-19 and PPADS significantly suppressed dynein activity and APL formation caused by high glucose. Taken together, these data suggest that ROS function as important players to regulate dynein-dependent AP trafficking leading to efficient autophagic maturation in CAMs.

## Introduction

*Autophagy* (also known as macroautophagy) is a *cellular* catabolic *pathway* leading to lysosomal *degradation* and recycling of *proteins and organelles* in eukaryotes 1. A crucial step during autophagic flux is autophagy maturation, a process of autophagosome (AP) trafficking and fusion with lysosomes to form autophagolysosomes (APLs) 2–4. Autophagy-related (Atg) genes have been identified to play essential roles for the formation of AP. However, the molecular mechanisms underlying autophagy maturation are relatively understudied. We recently demonstrated that dynein, a multi-subunit cytoplasmic motor protein, mediates AP trafficking, which contributes to the formation of APLs under pro-atherogenic stimulation in coronary arterial myocytes (CAMs) 5. However, it remains unknown how dynein’s function as a motor protein for AP trafficking is regulated in these vascular cells and whether such role of dynein is involved in autophagy maturation under other pathological conditions, such as hyperglycaemia.

Recently, reactive oxygen species (ROS) have been shown to interact with autophagy machinery 6. Increased generation of ROS and cellular oxidative stress by various stimuli serves as an important triggering mechanisms to induce autophagy 6–9. One mechanism for enhanced oxidative stress to trigger autophagy is through modulating Atg4 activity by oxidizing a critical cysteine residue in this protein 10. Atg4 cleaves LC3 to expose its C-terminal glycine to form conjugates with phosphatidylethanolamine by an ubiquitin-like system 11. Thus, redox regulation of Atg4 activity modulates the incorporation process of lipidated LC3 proteins into the autophagosomal membrane and subsequent AP formation as shown under starvation conditions 10. Reciprocally, ROS-activated autophagy pathway can protect cells from intensive oxidative stress by eliminating ROS-producing compartments, such as dysfunctional mitochondria 12. Thus, a complex interplay between ROS and autophagy pathways may exist under various pathological conditions, such as nutrition deprivation and hyperglycaemia. Despite ROS having been shown to regulate autophagy induction and AP formation at the early stage of autophagy, it remains elusive whether or how ROS modulate autophagy maturation, an event at the late stage of autophagy.

The present study tests the hypothesis that ROS may promote autophagy maturation *via* regulating dynein-mediated AP trafficking. Our data first demonstrated that ROS results in the activation of dynein ATPase. Then, we determined whether ROS-triggered AP trafficking and fusion with lysosomes is dependent on the activation of dynein ATPase, which is associated with NAADP-dependent lysosomal Ca^2+^ release. Lastly, we examined whether dynein-dependent AP trafficking is involved in autophagy maturation caused by high glucose–induced ROS production.

## Materials and methods

### Mice

Mice were purchased from the Jackson Laboratory (Bar Harbor, ME, USA). Eight-week-old male and female mice were used in all experiments. All experimental protocols were reviewed and approved by the Institutional Animal Care and Use Committee of Virginia Commonwealth University.

### Isolation and culture of mouse CAMs

Coronary arterial myocytes were isolated from mice as previously described 13. In brief, mice were deeply anaesthetized with an intraperitoneal injection of pentobarbital sodium (25 mg/kg). The heart was excised with an intact aortic arch and immersed in a petri dish filled with ice-cold Krebs–Henseleit (*KH*) solution (in mM: 20 HEPES, 128 NaCl, 2.5 KCl, 2.7 CaCl_2_, 1 MgCl_2_, 16 glucose, pH 7.4). A 25-gauge needle filled with Hank’s buffered saline solution (HBSS; in mM: 5.0 KCl, 0.3 KH_2_PO_4_, 138 NaCl, 4.0 NaHCO_3_, 0.3 Na_2_HPO_4_·7H_2_O, 5.6 D-glucose and 10.0 HEPES, with 2% antibiotics) was inserted into the aortic lumen while the whole heart remained in the ice-cold buffer solution. The tip of the needle was inserted deep into the heart near the aortic valve. The needle was tied in place with the needle tip as close to the base of the heart as possible. The infusion pump was started with a 20-ml syringe containing warm HBSS through an intravenous extension set at a rate of 0.1 ml/min. for 15 min. HBSS was replaced with a warm enzyme solution (1 mg/ml collagenase type I, 0.5 mg/ml soybean trypsin inhibitor, 3% BSA and 2% antibiotic–antimycotic), which was flushed through the heart at a rate of 0.1 ml/min. Perfusion fluid was collected at 30-, 60- and 90-min. intervals. At 90 min., the heart was cut with scissors and the apex was opened to flush out the cells that collected inside the ventricle. The fluid was centrifuged at 82 × g for 10 min., the cell-rich pellets were mixed with the one of the media described below, and the cells were plated on 2% gelatin-coated six-well plates and incubated in 5% CO_2_–95% O_2_ at 37°C. DMEM supplemented with 10% FBS, 10% mouse serum and 2% antibiotics was used to culture isolated smooth muscle cells. The medium was replaced 3 days after cell isolation and then once or twice each week until the cells grew to confluence. As previously described 14, mouse CAMs were identified according to their morphology, immunohistological staining, Western blot analysis of marker proteins and flow cytometric characteristics.

### Nucleofection of DCTN2 cDNA and Nox1 cDNA

Both dynamitin (Dynactin 2: DCTN2) cDNA (Catalog no. MC200162) and Nox1cDNA plasmids (Catalog no. MG226022) were purchased from OriGene Technologies. DCTN2 cDNA plasmid contains a full-length DCTN2 gene (1653 bp) under a cytomegalovirus (CMV) promoter. Nox1cDNA plasmid contains a full-length Nox1 gene (1692 bp) under a CMV promoter. Transfection of plasmid was performed with a 4D Nucleofector X-Unit (Lonza, Anaheim, CA, USA) according to the manufacturer’s instructions as we previously described 15. Briefly, CAMs were trypsinized and centrifuged at 90 × g for 10 min. The cell pellet was resuspended in 100 μl P1 Nucleofection solutions (Lonza) for Nucleofection (with the program code CM137). The program was chosen based on the fact that Nucleofection efficiency was over 80% as analysed by flow cytometry by using control GFP plasmids. For each Nucleofection sample, 2 μg plasmid DNA was added in 100 μl P1 Nucleofection solution. After Nucleofection, the cells were cultured in DMEM medium for 24 hrs and then were ready for treatment. The efficiency of DCTN2cDNA and Nox1cDNA transfection was assessed by Western blot analyses.

### Assay of cytoplasmic dynein ATPase activity

Dynein in mouse CAMs was purified by using a published protocol with slight modification 16. Cytoplasmic protein of mouse CAMs was extracted with ice-cold extraction buffer (250 ml of 0.05 M PIPES-NaOH, 0.05 M HEPES, pH 7.0, containing 2 mM MgC1_2_, 1 mM EDTA, 1 mM phenylmethylsulphonyl fluoride, 10 μg/ml leupeptin, 10 μg/ml tosyl arginine methyl ester, 1 μg/ml pepstatin A and 1 mM dithiothreitol (DTT). Exogenous taxol (20 μM) was added to 20 ml of cell extract containing 4 mg/ml cytoplasmic protein, which was incubated in a 37°C water bath (with occasional swirling) for 12 min. The cell extract was underlayered with a pre-warmed 7.5% sucrose solution, and then centrifuged at 60,000 × g for 30 min. at 35°C. The supernatant was removed and the pellet was resuspended in 10 ml of extraction buffer containing 3 mM MgGTP and 5 μM taxol to release kinesin and dynamin. The resuspended pellet was incubated for 15 min. prior to centrifugation at 60,000 × g for 30 min. The supernatant was removed, and the pellet was resuspended in 1.25 ml of extraction buffer containing 10 mM Mg-ATP for 10 min. at 37°C. The resuspended pellet was centrifuged at 200,000 × g for 30 min. at 25°C. The supernatant containing ATP-released cytoplasmic dynein was used for sucrose density gradient fractionation. Cytoplasmic dynein may constitute up to 50% of total protein in the ATP extract, the remainder consisting of tubulin and a low level of fibrous microtubule-associated proteins (MAPs). One millilitre of ATP extract was further centrifuged on 10 ml of a 5–20% sucrose gradient in fractionation buffer (20 mM Tris-HCl, pH 7.6, 50 mM KCl, 5 mM MgSO_4_, 0.5 mM EDTA and 1 mM DTT) at 125,000 × g for 16 hrs at 4°C. Eleven 1-ml fractions were collected from the bottom of the tube. The dynein fraction peak was at about fraction 5, well resolved from the other tubulin and MAPs.

The assays of dynein ATPase activity were performed in 50 μl reaction mixtures containing 20 mM Tris-HCl (pH 7.6), 50 mM KCl, 5 mM MgSO_4_, 0.5 mM EDTA and 1 mM DTT 17. In a standard assay condition, 10 μl of enzyme fractions and 4 mM of ATP were incubated with assay buffer at 37°C for 40 min. The reaction was then stopped by using highly acidic malachite green reagent, and the absorbance was read at 660 nm in spectrophotometer (Elx800; Bio-Tek, Winooski, VT, USA). The amount of inorganic phosphate release in the enzymatic reaction was calculated by using the standard calibration curve generated with inorganic phosphate. The control in this assay contained all ingredients of the reaction mixture, but the reaction was stopped at 0 time.

### Dynamic analysis of AP movement in CAMs

Coronary arterial myocytes (2 × 10^4^/ml) cultured in a 35-mm dish were incubated with 12 μl BacMam GFP-LC3B virus particles at 37°C for 16 hrs to express the LC3B-GFP gene. The confocal fluorescent microscopic recording was conducted with an Olympus Fluoview System. The fluorescent images for AP (LC3B-GFP) in the CAMs were continuously recorded at an excitation/emission (nm) of 485/520 by using the XYT recording mode with a speed of 1 frame/10 sec. for 10 min. Vesicle tracking was performed in MAGEJ by using the LSM reader and Manual tracking plug-ins according to the published protocol 18. Ten vesicles with GFP-LC3B were chosen at random for each cell. These vesicles were then tracked manually for as long as they were visible, while the program calculated velocities for each frame. All the results were further calculated and analysed in Excel. The number of cells with different velocity of AP was calculated.

### Western blot analysis

Western blot analysis was performed as we described previously 15. In brief, proteins from the CAMs were extracted by using sucrose buffer [20 mM HEPES, 1 mM EDTA, 255 mM sucrose, cocktail of protease inhibitors (Roche, Nutley, NJ, USA), pH 7.4]. After boiling for 5 min. at 95°C in a 5× loading buffer, 30 μg of total proteins was separated by a 12% SDS-PAGE. The proteins of these samples were then electrophoretically transferred at 100 V for 1 hr onto a PVDF membrane (Bio-Rad, Hercules, CA, USA). The membrane was blocked with 5% non-fat milk in Tris-buffered saline-Tween 20. After washing, the membrane was probed with 1:1000 dilution of primary mouse or rabbit antibodies against DCTN2 (Santa Cruz, Dallas, TX, USA), Nox1 (Santa Cruz), Lamp1 (Novus, Littleton, CO, USA) or β-actin (Santa Cruz) overnight at 4°C followed by incubation with horseradish peroxidase-labelled IgG (1:5000). The immunoreactive bands were detected by chemiluminescence methods and visualized on Kodak Omat X-ray films. Densitometric analysis of the images obtained from X-ray films was performed with the Image J software (NIH, Littleton, CO, USA).

### Confocal microscopic analysis of co-localization of lysosomes with AP in live CAMs

To monitor the fusion of AP with lysosomes in live CAMs, AP and lysosomes were labelled with fluorescent proteins by introducing LC3B-GFP and Lamp1-RFP genes into CAMs. To this end, BacMam (baculovirus-based expression in mammalian cells) expression system was utilized to deliver and express LC3B-GFP and Lamp1-RFP genes in CAMs. Modified insect virus (baculovirus) expressing a fusion construct of LC3B-GFP (P36235; Invitrogen, Grand Island, NY, USA) or Lamp1-RFP (C10597; Invitrogen) was packaged as BacMam virus particle and purchased from Invitrogen. Briefly, CAMs (4 × 10^4^/ml) cultured in 35-mm dish were incubated with 12 μl mixture of BacMam virus particles containing LC3B-GFP or Lamp1-RFP gene at 37°C for 24 hrs. Then, the cells were replaced with fresh medium and ready for treatment. The fluorescent images for AP (LC3B-GFP) and lysosomes (Lamp1-RFP) in CAMs were recorded at an excitation/emission (nm) of 485/520 and 555/584. Then the co-localizations were visualized with confocal microscopy. The co-localization coefficient of LC3B-GFP and Lysosomes-RFP was analysed with Image-Pro Plus 6.0 software as we previously described 19.

### Flow cytometric detection of AP and APLs

The Cyto-ID Autophagy Detection Kit (Enzo Life Sciences, Farmingdale, NY, USA) was used to detect AP 5. Briefly, CAMs (1 × 10^5^/ml) were collected and centrifuged (400 × g, 5 min.) at the end of the treatment. Then, CAMs were incubated with 0.5 ml of freshly diluted Cyto-ID Green Detection Reagent (1:4000) for 30 min. at 37°C in the dark. Without washing, stained CAMs were run in the green (FL1) channel with a Guava Easycyte Mini Flow Cytometry System (Guava Technologies, Hayward, CA, USA) and analysed with Guava acquisition and analysis software (Guava Technologies). The enhancement of Cyto-ID Green dye signal indicates an increase in AP.

In addition, acridine orange (Sigma-Aldrich, St. Louis, MO, USA) was used to detect APLs. CAMs (1 × 10^6^/ml) were stained with acridine orange (1:5000) for 17 min. After washes, CAMs were harvested in phenol red-free growth medium. Green (510–530 nm) and red (>650 nm) fluorescence emission from 10^4^ cells illuminated with blue (488 nm) excitation light was measured with Flow Cytometry System and analysed with Guava acquisition and analysis software. The mean red/green fluorescence ratio was calculated to indicate the change of intracellular APLs.

### Assays for lysosomal pH

To observe lysosomal alkalinization, we incubated CAMs in PBS buffer with or without 100 μM chloroquine for 30 min. The cells were then loaded with LysoSensor Green DND-189 (1 μM; Invitrogen) in PBS buffer for 15 min. at 37°C. Cells were washed twice with PBS and immediately visualized with a confocal laser scanning microscope (Fluoview FV1000; Olympus, Tokyo, Japan).

### Confocal microscopic analysis of co-localization of ubiquitin and p62 in CAMs

For confocal analysis, cultured CAMs were grown on glass coverslips, stimulated or unstimulated, fixed in 4% paraformaldehyde in phosphate-buffer saline (PFA/PBS) for 15 min. After being permeabilized with 0.1% Triton X-100/PBS and rinsed with PBS, the cells were incubated overnight at 4°C with indicated primary antibodies: mouse anti-ubiquitin and rabbit anti-p62 (1:200; Cell Signaling, Danvers, MA, USA). After washing, the slides were probed with primary antibodies and were then incubated with Alexa-488- or Alexa-555-labelled secondary antibodies for 1 hr at room temperature. The slides were mounted and subjected to examinations by using sequential scanning on a laser scanning confocal microscope (Fluoview FV1000; Olympus), with photographs being taken and the co-localization analysed by the Image-Pro Plus 6.0 software (Media Cybernetics, Bethesda, MD, USA). The summarized co-localization efficiency data were expressed as Pearson correlation coefficient (PCC) as described previously 19.

### Fluorescent microscopic measurement of [Ca^2+^]_i_ in CAMs

A fluorescence image analysis system was used to determine intracellular Ca^2+^ concentration ([Ca^2+^]_i_) in CAMs with fura-2 acetoxymethyl ester (fura-2) as an indicator as previously described 14,20. Having been loaded with 10 μM fura-2 at room temperature for 30 min., the cells were washed three times with Ca^2+^-free Hank’s buffer. The ratio of fura-2 emissions, when excited at the wavelengths of 340 and 380 nm, was recorded with a digital camera (Nikon Diaphoto TMD Inverted Microscope). Metafluor imaging and analysis software were used to acquire, digitize and store the images for offline processing and statistical analysis (Universal Imaging, Bedford Hills, NY, USA). The fluorescence ratio of excitation at 340 nm to that at 380 nm (F340/F380) was determined after background subtraction, and [Ca^2+^]_i_ was calculated by using the following equation: [Ca^2+^]_i_ = *K*_d_β[(R − R_min_)/(R_max_ − R)], where *K*_d_ for the fura-2-Ca^2+^ complex is 224 nM; R is the fluorescence ratio (F_340_/F_380_); R_max_ and R_min_ are the maximal and minimal fluorescence ratios measured by addition of 10 μM of Ca^2+^ ionophore ionomycin to Ca^2+^-replete solution (2.5 mM CaCl_2_) and Ca^2+^-free solution (5 mM EGTA) respectively; and β is the fluorescence ratio at 380-nm excitation determined at R_min_ and R_max_ respectively. Lysosomal Ca^2+^ release was monitored indirectly by treating Fura-2-loaded CAMs with Glycyl-L-phenylalanine 2-naphthylamide (GPN, 200 μM), a tripeptide causing osmotic lysis of cathepsin C-positive lysosomes.

### Confocal microscopic measurement of lysosome Ca^2+^ release

To detect lysosome Ca^2+^ release, subconfluent CAMs in 35-mm cell culture dishes were incubated with dextran-conjugated tetramethylrhodamine (Rho; 1 mg/ml; Molecular Probes, Life Technologies, Grand Island, NY, USA) for 4 hrs in DMEM medium containing 10% FBS at 37°C, 5% CO_2_ followed by a 20-hr chase in dye-free medium for lysosomes loaded with Rho, as previously described 21,22. After being washed with HBSS (in mM: 5.0 KCl, 0.3 KH_2_PO_4_, 138 NaCl, 4.0 NaHCO_3_, 0.3 Na_2_HPO_4_·7H_2_O, 5.6 D-glucose and 10.0 HEPES, with 2% antibiotics) three times, the Rho-loaded cells were then incubated with the Ca^2+^-sensitive dye fluo-4 at a concentration of 5 μM. Ca^2+^ release and lysosome trace recordings were performed. Lysosome/Rho (Lyso/Rho) fluorescence images were acquired at 568-nm excitation and 590-nm emission. The co-localization coefficiency of Ca^2+^/fluo-4 and Lyso/Rho was analysed with Image-Pro Plus 6.0 software 23.

### Statistics

Data are presented as means ± SE. Significant differences between and within multiple groups were examined by using anova for repeated measures, followed by Duncan’s multiple-range test. The Students *t*-test was used to detect significant differences between two groups. *P* < 0.05 was considered statistically significant.

## Results

### ROS enhances dynein ATPase activity in CAMs

**Figure 1:**
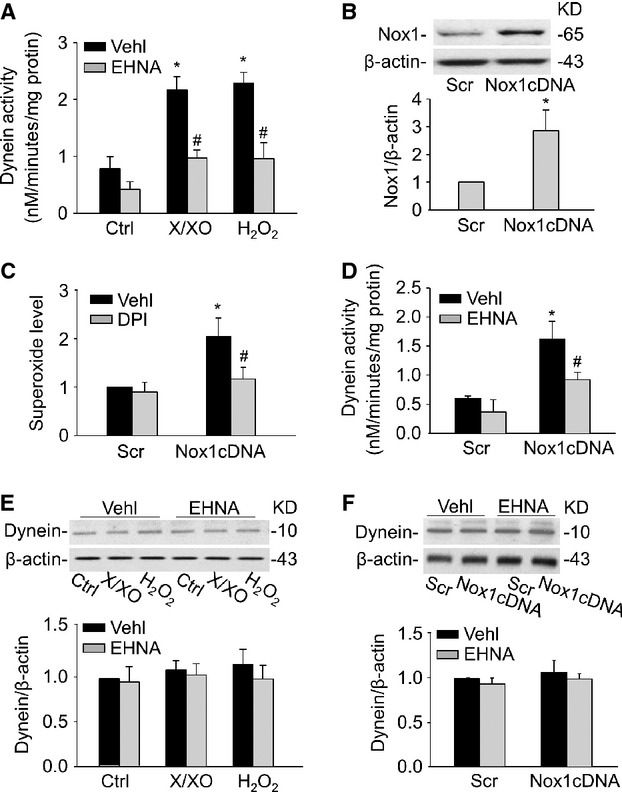
Regulation of dynein ATPase activity by ROS. (**A**) CAMs were incubated with X/XO (10 μM/0.1 U/ml) or H_2_O_2_ (10 μM) for 24 hrs in the presence or absence of dynein inhibitor EHNA (30 μM). Summarized data showing the dynein ATPase activity induced by X/XO or H_2_O_2_. (**B**) Representative Western blot gel document and summarized intensity ratio of Nox1 to β-actin showing the expression of Nox1 in CAMs with scramble (Scr) or Nox1 cDNA plasmid transfection. (**C**) Summarized ESR data showing the relative O_2_^−.^ production in CAMs with scramble or Nox1 cDNA transfection in the absence or presence of DPI (50 μM). (**D**) Summarized data showing the effects of dynein inhibitor EHNA (30 μM) on dynein ATPase activity in CAMs with scramble or Nox1 cDNA transfection. (**E** and **F**) Representative Western blot gel document and summarized data showing the protein expression of dynein in CAMs under X/XO or H_2_O_2_ stimulation (**E**) and after Nox1 cDNA transfection (**F**; *n* = 6 for all panels). *P < 0.05 *versus* Ctrl or Scramble; ^#^*P* < 0.05 *versus* CAMs treated with X/XO or H_2_O_2_ or Nox1 cDNA transfection alone.

Dynein is a motor protein responsible for nearly all minus-end microtubule-based transport of vesicles in eukaryotic cells and has recently been implicated in AP trafficking and fusion with lysosomes to form APLs 5. Superoxide (O_2_^−.^) and hydrogen peroxide (H_2_O_2_) are the major ROS implicated in regulating autophagy 6. Here, we demonstrated that treatment of CAMs with xanthine/xanthine oxidase (X/XO), a typical O_2_^−.^ production system, or H_2_O_2_ resulted in a marked increase in dynein ATPase activity, which was significantly attenuated by EHNA, a dynein activity inhibitor (Fig. 1A).

NADPH oxidase-generated ROS contribute to autophagy induction 24–26. To examine whether endogenous ROS produced by NADPH oxidase have a similar effect as exogenous ROS on dynein activity, CAMs were transfected with NADPH oxidase isoform 1 (Nox1) cDNA plasmids. Nox1 cDNA transfection increased protein expression of Nox1 by 2.85-fold (Fig. 1B). Consistently, O_2_^***−***.^ production in CAMs was significantly increased after the Nox1 cDNA transfection, which was inhibited by NADPH oxidase inhibitor, diphenyleneiodonium (*DPI*; Fig. 1C). Similar to exogenous ROS, overexpression of Nox1 increased production as well as dynein ATPase activity in CAMs (Fig. 1D). However, both exogenous and Nox1-derived ROS had no effect on the protein expression of dynein. Moreover, there was no significant change of dynein expression after the pre-treatment with EHNA, which showed that inhibition of dynein function has no effects on the expressions of dynein (Fig. 1E and F).

### Dynamic analysis of AP movement upon ROS stimulation

**Figure 2:**
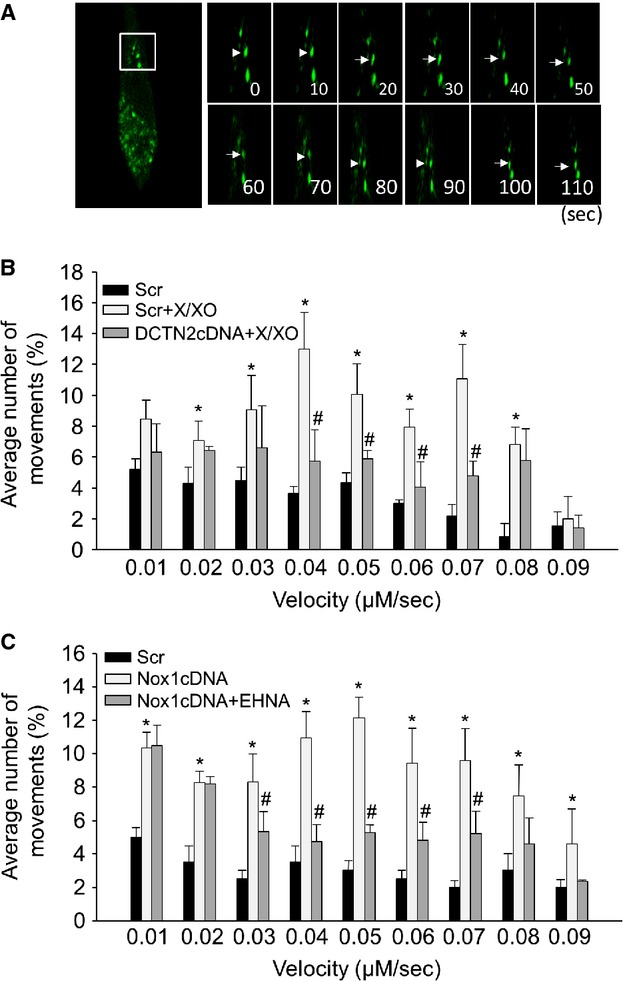
Analysis of AP dynamic movements. (**A**) Typical fluorescent images of LC3B-GFP-labelled AP in CAMs were taken every 10 sec. in CAMs. (**B** and **C**) The summarized data show the velocity of AP in CAMs pre-treated with EHNA (30 μM) or transfected with dynamitin (DCTN2) cDNA (*n* = 6 for all panels). *P < 0.05 *versus* Scramble; ^#^*P* < 0.05 *versus* CAMs with X/XO or Nox1 cDNA transfection alone.

Next, we examined whether AP trafficking is a consequence of ROS-sensitive dynein activity. AP are formed randomly throughout the cytoplasm and then transported to the perinuclear region where they fuse with lysosomes 18. Microtubule-associated protein 1 light chain 3 beta (LC3B) is a marker protein of AP, which is recruited to autophagosomal membranes during the formation of AP. We labelled AP in living CAMs with LC3B-GFP and monitored their movements upon ROS treatment. Typically, fluorescent images of CAMs were taken every 10 sec. (Fig. 2A). Under control condition, AP moved bidirectionally, *i.e*. towards and away from the nucleus. When CAMs were treated with X/XO, the movement of AP was significantly enhanced (Fig. 2B). The velocity of AP movement significantly increased from 0.03 mm/sec. to 0.08 mm/sec. Similar effects were also found in H_2_O_2_-treated CAMs (data not shown). This ROS-induced AP movement (velocity range between 0.04 mm/sec. and 0.07 mm/sec.) was completely inhibited in CAMs with DCTN2 overexpression, which causes disruption of the dynein complex. Similarly, endogenous ROS production by Nox1 overexpression increased the velocity of AP movement, which was inhibited by dynein inhibitor EHNA (Fig. 2C).

### Dynein contributes o ROS-induced AP fusion to lysosome

**Figure 3:**
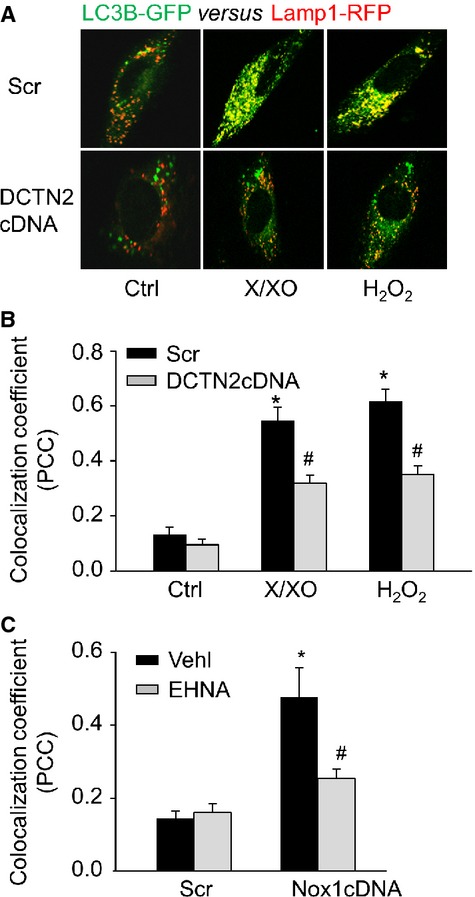
Inhibition of dynein activity blocked lysosome fusion. (**A**) Representative confocal microscopic images showing the co-localization of LC3B-GFP with Lamp1-RFP in live CAMs. (**B**) Summarized co-localization coefficient (PCC) of LC3B and Lamp1 in CAMs transfected with scramble or DCTN2 cDNA. (**C**) Summarized co-localization coefficient of LC3B-GFP and Lamp1-RFP in CAMs with or without EHNA (30 μM; *n* = 6 for all panels). *P < 0.05 *versus* Ctrl or Scramble; ^#^*P* < 0.05 *versus* CAMs treated with X/XO or H_2_O_2_ or Nox1 cDNA transfection alone.

To directly observe the role of ROS-sensitive dynein activity on the fusion of AP with lysosomes in living cells, both LC3B-GFP and Lamp1-RFP (Lamp-1 is a lysosome marker protein) genes were introduced into CAMs to visualize AP (green puncta) and lysosomes (red puncta). Fusion of AP with lysosomes increases the co-localization of LC3B-GFP with Lamp1-RFP and yields yellow puncta. As shown in Figure 3A and B, under control conditions, only a few yellow puncta were detected. Both X/XO and H_2_O_2_ treatment markedly increased the number of yellow puncta and the co-localization coefficient between LC3B-GFP and Lamp1-RFP indicating a higher fusion rate of lysosomes with AP. However, such ROS-induced AP–lysosome fusion was inhibited by DCTN2 overexpression. Similarly, Nox1 cDNA transfection markedly enhanced the AP–lysosome fusion, which was inhibited by dynein inhibitor EHNA (Fig. 3C).

### Dynein is involved in the ROS-induced APLs formation

**Figure 4:**
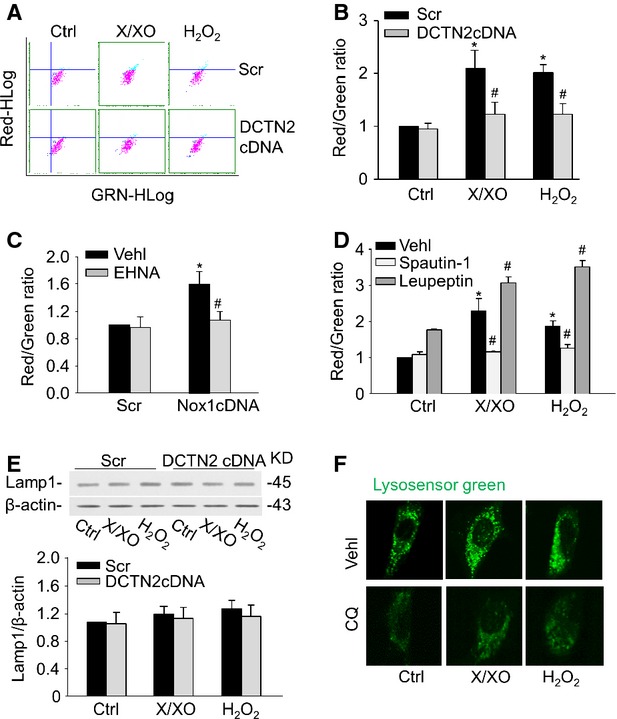
Inhibition of dynein activity decreased APLs formation. Mouse CAMs were stained with acridine orange for 17 min. Representative dot plots of flow cytometry (**A**) and summarized red-to-green fluorescence ratio analysis showing APLs formation in CAMs with DCTN2 cDNA (**B**) or EHNA (**C**). (**D**) Summarized red-to-green fluorescence ratio analysis showing APLs formation in CAMs with spautin-1 (10 μM) and leupeptin (0.25 mM). (**E**) Representative Western blot documents showing the expression of Lamp-1 from CAMs. (**F**) CAMs were treated with chloroquine (CQ, 100 μM) for 30 min. or left untreated. They were then stained with LysoSensor Green DND-189 and analysed by fluorescence microscopy (*n* = 6 for all panels). *P < 0.05 *versus* Scramble; ^#^*P* < 0.05 *versus* CAMs with X/XO or H_2_O_2_ or Nox1 cDNA transfection alone.

The formation of APLs was also quantified by using flow cytometry with a lysomotrophic dye, acridine orange, which accumulates in lysosomes with bright red fluorescence and shows bright green and dim red fluorescence in the cytoplasm and nucleolus. As APLs accumulate more acridine orange than lysosomes, the red/green fluorescence ratio indirectly measures the change of intracellular APLs 27. Figure 4A shows that CAMs treated with X/XO or H_2_O_2_ shifted up to the area with high red fluorescence intensity. Quantification of the data in Figure 4B indicated that X/XO or H_2_O_2_ significantly increased the red/green fluorescence ratio suggesting that APLs formation was increased, and these effects were abrogated by DCTN2 overexpression. Similarly, inhibition of dynein activity by EHNA markedly attenuated APLs formation induced by Nox1 overexpression (Fig. 4C). Consistently, ROS-induced APLs formation was inhibited by spautin-1, a potent small molecule inhibitor of AP formation (Fig. 4D). In contrast, ROS-induced APLs formation was further enhanced by leupeptin, a lysosomal protease inhibitor that blocks APLs degradation without changing lysosomal pH (Fig. 4D). This result indicates that ROS indeed increase the formation of APLs rather than inhibit their degradation. Increased AP–lysosome fusion by ROS may be associated with enhanced lysosome biogenesis or acidification. However, ROS had no effects on Lamp1 expression (Fig. 4E) or lysosomal pH (Fig. 4F), ruling out these possibilities. Moreover, DCTN2 overexpression had no effects on the expression of Lamp-1, which showed that inhibition of dynein function has no effects on the expressions of Lamp-1.

### Dynein-regulated AP accumulation and autophagic degradation upon ROS stimulation

**Figure 5:**
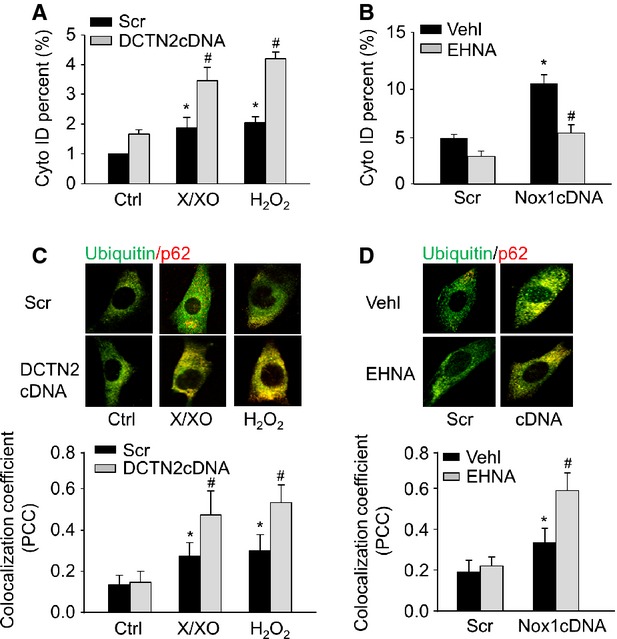
Dynein inhibition increased accumulation of AP and decreased breakdown of AP. (**A** and **B**) Summarized per cent of Cyto-ID-stained cells showing the relative number of AP in CAMs with EHNA or DCTN2 cDNA. (**C** and **D**) Representative confocal images and summarized co-localization coefficient showing the co-localization of ubiquitin with p62 (*n* = 6 for all panels). *P < 0.05 *versus* Ctrl or Scramble; ^#^*P* < 0.05 *versus* CAMs treated with X/XO or H_2_O_2_ or Nox1 cDNA transfection alone.

The role of dynein on ROS-induced autophagy maturation was further investigated by evaluating the effects of dynein inhibition on AP accumulation and autophagic degradation. A Cyto-ID Green dye was used to selectively label AP, and the percentage of Cyto-ID-positive cells that correlates with the number of AP was analysed by flow cytometry 28. Figure 5A showed that X/XO or H_2_O_2_ increased the percentage of Cyto-ID-positive cells indicating that more AP were formed. Such increases were further augmented by dynein inhibitor EHNA suggesting that the loss of dynein function results in AP accumulation under ROS stimulation. Inclusion of granular ubiquitin or selective autophagy substrate p62 in AP of cells can be used to detect the breakdown of autophagic vesicles 29. As shown in Figure 5C, compared with scramble-transfected cells, CAMs transfected with DCTN2 cDNA exhibited much more yellow puncta and increased co-localization between ubiquitin and p62 upon X/XO or H_2_O_2_ treatment. This suggests a failed breakdown of AP in the absence of dynein activity. Similar results were found in CAMs transfected with Nox1 cDNA when dynein was inhibited by EHNA (Fig. 5B and D).

### ROS-induced NAADP-sensitive Ca^2+^ release

**Figure 6:**
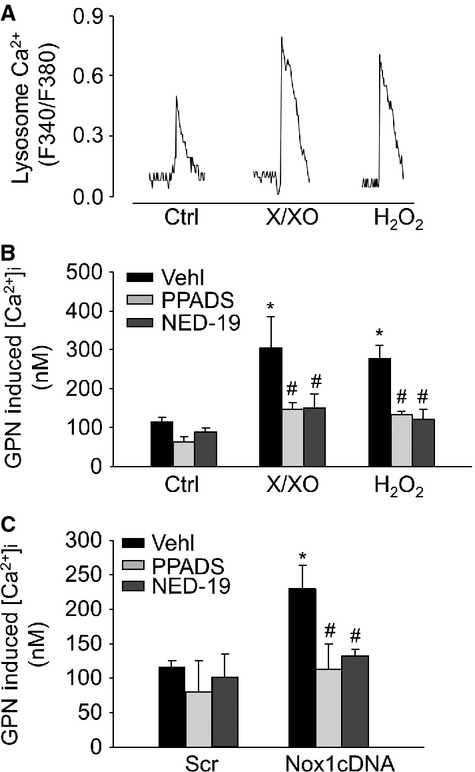
Lysosome Ca^2+^ release in response to ROS in living CAMs. Glycyl-l-phenylalanine-β-naphthylamide (GPN, 200 μM) was used to release Ca^2+^ from lysosomes. Representative lysosomal Ca^2+^ traces (**A**) and summarized data showing the effects of NAADP antagonists, PPADS (50 μM) or NED-19 (10 μM) on GPN-induced lysosomal Ca^2+^ release in CAMs treated with X/XO or H_2_O_2_ (**B**) or Nox1 cDNA (**C**; *n* = 6 for all panels). *P < 0.05 *versus* Ctrl or Scramble; ^#^*P* < 0.05 *versus* CAMs treated with X/XO or H_2_O_2_ or Nox1 cDNA transfection alone.

NAADP-sensitive lysosomal Ca^2+^ release has been implicated in the regulation of dynein activity under proatherogenic stimulation 5. To test whether lysosomal Ca^2+^ was involved in ROS-induced APs trafficking, fluorescent imaging analysis was used to measure the lysosomal Ca^2+^ release in CAMs. GPN was used to release Ca^2+^ from lysosomes by inducing their selective osmotic swelling. Both X/XO and H_2_O_2_ significantly increased GPN-induced Ca^2+^ release, which was blocked by NAADP antagonists NED-19 and PPADS (Fig. 6A and B). Similar results were also found in CAMs transfected with Nox1cDNA (Fig. 6C).

**Figure 7:**
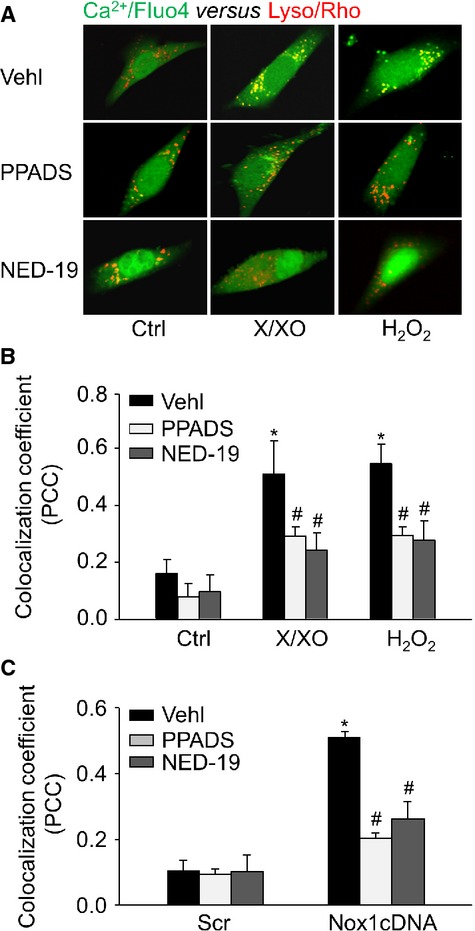
Confocal microscopic detection of Ca^2+^ release from lysosomes locally in CAMs. (**A**) Representative confocal microscopy images showing Ca^2+^ release regions that co-localized with lysosomes as shown by yellow spots formed by green fluo-4 signals with rhodamine-red lysosomal marker (Lyso/Rho). Summarized data showing the co-localization coefficient of Ca^2+^/fluo-4 with Lyso/Rho signals in CAMs treated with X/XO or H_2_O_2_ (**B**) or Nox1 cDNA (**C**; *n* = 6 for all panels). *P < 0.05 *versus* Ctrl or Scramble; ^#^*P* < 0.05 *versus* CAMs treated with X/XO or H_2_O_2_ or Nox1 cDNA transfection alone.

In addition, we measured the localization of Ca^2+^ around lysosomes by confocal microscopy after labelling cells with fluo-4 and rhodamine-red (Lyso/Rho) lysosomal marker. Ca^2+^ release regions co-localized with lysosomes as indicated by yellow spots formed by the close proximity of green fluo-4 signals and rhodamine-red (Fig. 7A). Both X/XO and H_2_O_2_ significantly increased the co-localization coefficient of fluo-4-Ca^2+^ and Lyso/Rho, indicating an enhanced lysosomal Ca^2+^ release, while NAADP antagonists PPADS or NED-19 abolished these effects (Fig. 7B). Similarly, enhanced lysosomal Ca^2+^ release in CAMs by Nox1 overexpression was blocked by NAADP antagonists (Fig. 7C).

### Lysosomal Ca^2+^ release regulates dynein ATPase activity in CAMs upon ROS stimulation

**Figure 8:**
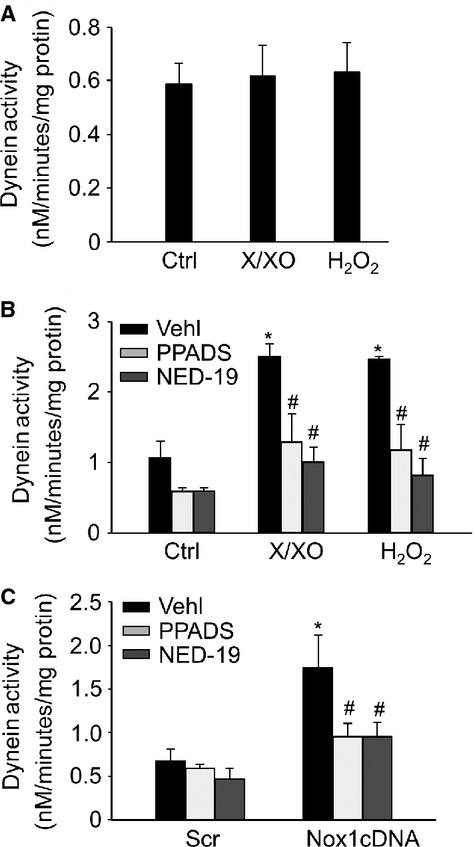
Lysosome Ca^2+^ signalling-regulated dynein ATPase activity. (**A**) Ten microgram proteins of isolated cytoplasmic lysates from CAMs was incubated with X/XO (10 μM/0.1 U/ml) or H_2_O_2_ (10 μM) for 2 hrs. Summarized data showing the direct oxidizing effects of ROS on dynein ATPase activity. (**B** and **C**) Summarized data showing the effects of NAADP antagonists, PPADS (50 μM) or NED-19 (10 μM) on dynein ATPase activity in CAMs under X/XO (10 μM/0.1 U/ml) or H_2_O_2_ (10 μM) stimulation or Nox1 cDNA transfection in CAMs (*n* = 6 for all panels). *P < 0.05 *versus* Ctrl or Scramble; ^#^*P* < 0.05 *versus* CAMs treated with X/XO or H_2_O_2_ or Nox1 cDNA transfection alone.

To determine how ROS regulate dynein ATPase activity, we first examined whether ROS can directly oxidize dynein to enhance its activity. To this end, the extracted cytoplasmic protein lysates from CAMs were incubated with X/XO and H_2_O_2_. Surprisingly, X/XO and H_2_O_2_ had no direct effects on dynein ATPase activity (Fig. 8A). We then examined whether NAADP-mediated lysosomal Ca^2+^ signalling is an upstream event of ROS-enhanced dynein activation. Indeed, ROS-enhanced dynein ATPase activation was significantly attenuated when the intact CAMs were treated with NAADP antagonists NED-19 and PPADS (Fig. 8B and C).

### Nox1-regulated dynein activity mediates APLs formation under high glucose

**Figure 9:**
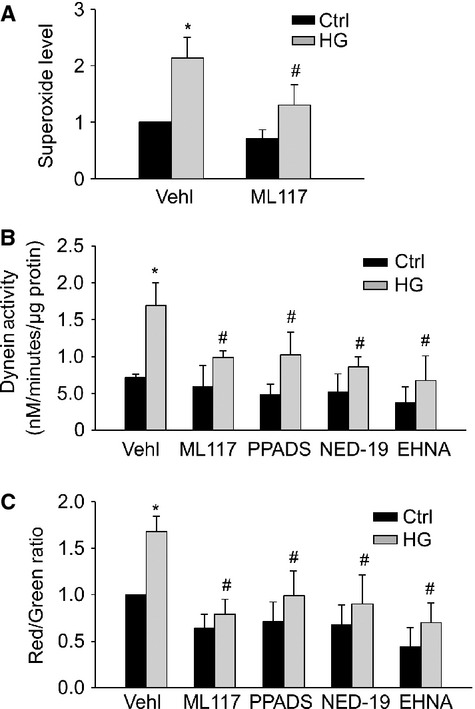
NAADP-lysosome Ca^2+^-controlled high glucose–induced dynein activation. (**A**) Summarized data showing the effects of Nox1 inhibitor ML117 (100 μM) on O_2_^−^ production in CAMs under control and high glucose (30 mM) for 48 hrs. Summarized data showing the effects of Nox1 inhibitor ML117, dynein inhibitor EHNA and NAADP antagonists PPADS or NED-19 on dynein ATPase activity (**B**) or APLs formation (red-to-green fluorescence ratio of acridine orange; **C**) in CAMs under resting control condition or with high glucose (*n* = 6 for all panels). *P < 0.05 *versus* Ctrl; ^#^*P* < 0.05 *versus* CAMs treated with high glucose.

Finally, we examined whether ROS-sensitive dynein ATPase controls autophagy maturation under high-glucose conditions. High-glucose treatment induces ROS production in vascular smooth muscle cells *via* activation of Nox1 30. Consistently, treatment of CAMs with high glucose (30 mM) resulted in a marked increase of O_2_^−.^ that was inhibited by ML117, a Nox1-specific inhibitor (Fig. 9A). Moreover, high glucose treatment significantly increased dynein ATPase activity in CAMs, which was significantly attenuated by ML117 or NAADP antagonists, NED-19 or PPADS (Fig. 9B). Importantly, the increased APLs formation, as shown by increased red/green fluorescence ratio of acridine orange staining under *high glucose* treatment, was abolished by pre-treatment of CAMs with Nox1 inhibitor ML117, NAADP antagonists NED-19 or PPADS, or dynein inhibitor EHNA (Fig. 9C).

## Discussion

The present study demonstrates that in CAMs, ROS enhance the activity of dynein ATPase, a microtubule motor protein, which promotes AP trafficking and fusion with lysosomes to form APLs contributing to autophagy maturation. NAADP-dependent lysosomal Ca^2+^ release plays a critical role in controlling dynein-mediated autophagy maturation under either redox or high glucose condition.

Reactive oxygen species are rapidly produced, short-lived and diffusible reactive molecules and are traditionally considered as cellular damage factors. However, accumulating evidence suggests that under physiological conditions or in the early stage of oxidative damage, ROS also act as signalling molecules to mediate cellular responses such as autophagy by inducing AP formation 31. Such role of ROS in autophagy is attributed to their interactions with Atg proteins such as oxidative regulation of Atg4, a crucial factor mediating LC3 lipidation 9. However, the molecular mechanisms underlying the ROS-triggered maturation of AP to APLs are relatively unknown. Here, we demonstrated that treatment of mouse CAMs with ROS or overexpressing Nox1, a ROS-producing NADPH oxidase isoform, in these cells dramatically enhanced the dynein ATPase activity indicating that dynein could be a potential target protein for ROS to modulate autophagy maturation.

During autophagy maturation, *de novo* generated AP sequester cytoplasmic proteins and organelles, which are delivered to lysosomes for degradation 32. In this process, AP show a rapid vectorial movement to the direction of the centrosome, where lysosomes are usually concentrated 18. Dynein is a microtubule-associated motor protein involved in AP trafficking in mammalian cells 2,5,18,33,34. Inhibition or loss of dynein function impairs AP trafficking in glioma or neuronal cells 35. The present study demonstrated that both exogenous and Nox1-derived ROS increased the movement of AP in CAMs, which was inhibited by either dynein inhibitor EHNA or disruption of the dynein complex with dynamitin (gene symbol is DCTN2) overexpression. These data suggest that dynein-mediated AP trafficking is also regulated by ROS. As AP trafficking promotes AP fusion with lysosomes, we further observed increased co-localization of AP with lysosomes induced by ROS by using LC3B-GFP and Lamp1-RFP transfected CAMs (Fig. 3). In addition, increased fusion of LC3B-GFP with Lamp1-RFP by ROS was confirmed by showing increased APLs formation by ROS by using acridine orange staining (Fig. 4A–D). As a consequence of impaired AP trafficking, both ROS-induced AP–lysosome fusion and APLs formation were blocked by inhibition of dynein or disruption of dynein complex. Thus, our data suggest that redox regulation of the dynein activity controls autophagy maturation through AP trafficking and fusion with lysosomes.

Autophagy maturation results in the formation of functional APLs that break down their autophagic contents and ultimately themselves by lysosomal proteases. Inhibition of dynein function increased AP accumulation in glioma and neuronal cells 35, which indicates that accumulated AP as a result of an impaired autophagic degradation pathway are a characteristic of insufficient autophagy maturation. In the present study, we directly monitored the number of AP by flow cytometry by using Cyto-ID Green probes and found that ROS-induced AP formation was further augmented by either EHNA or dynamitin overexpression indicating more AP were accumulated by ROS in the absence of dynein function. During autophagy, the p62 protein, an ubiquitin-binding scaffold protein, targets ubiquitinated protein aggregates to AP for autophagic degradation 29. Thus, we also detected the breakdown of autophagic vesicles by determining the inclusion of granular ubiquitin or p62 in AP 36. Our data revealed that ROS treatment resulted in more ubiquitin and p62 in AP with dynein inhibition suggesting that a failed breakdown of autophagic vesicles leads to AP accumulation. Together, these results further support the view that dynein-mediated AP trafficking and fusion with lysosomes contributes to ROS-induced autophagy maturation leading to an efficient autophagic degradation pathway.

Another important finding of the present study is the critical role of lysosomal Ca^2+^ release machinery in mediating ROS-induced dynein activation. In present study, we first demonstrated that ROS did not regulate dynein ATPase activity *via* direct oxidization of its subunits or interacting proteins in a cell-free system (extracted cytoplasmic proteins from CAMs were treated with ROS; Fig. 8A). Previous studies have reported that direct binding of Ca^2+^ to dynein complex regulates dynein motor function and the distribution of cytoplasmic dynein 37,38. *NAADP, a CD38-ADP-ribosylcyclase product, is one of the most potent intracellular Ca^2+^*-*mobilizing molecules, which regulates lysosomal* Ca^2+^ release and lysosome function 39–41. Previous study had demonstrated that exogenous O_2_^−.^ increase ADP-ribosylcyclase activity, cADPR production, leading to mobilization of intracellular Ca^2+^ from the SR in mouse CAMs 42. Recently, it was demonstrated that endogenous Nox1-derived O_2_^−.^ serves in an autocrine fashion to enhance CD38 internalization, leading to redox activation of CD38/ADP-ribosylcyclase activity and NAADP production 40. Thus, these previous studies suggest that ROS-induced NAADP signalling is initiated *via* redox activation of CD38/ADP-ribosylcyclase. In the present study, NAADP antagonists NED-19 or PPADS markedly reduced ROS-induced augmentation of lysosomal Ca^2+^ release and dynein activity in CAMs. Therefore, our data suggest that ROS function as signalling molecules to regulate dynein activity *via* enhancing NAADP-mediated lysosomal Ca^2+^ release.

The present study further revealed a critical role of dynein-mediated AP trafficking machinery in autophagy maturation associated with oxidative stress under pathological conditions, such as high glucose. Hyperglycaemia is a major causative factor in the development of diabetes-associated diseases. NADPH oxidase-associated oxidative stress has been identified as an important mechanism in the pathogenesis of hyperglycaemia-induced vascular damage 43. Previous studies revealed that Nox1 is a major NADPH oxidase isoform responsible for high glucose–induced O_2_^−.^ production in vascular smooth muscle cells 30. We consistently demonstrated that specific Nox1 inhibitor ML117 abolished high glucose–induced O_2_^−.^ production, dynein activation and APLs formation. In addition, NAADP antagonists and dynein inhibitor mimicked the effects of ML117 on APLs formation; therefore, our data suggest that Nox1-derived ROS/NAADP-Ca^2+^/dynein axis is involved autophagy maturation under high glucose condition in CAMs. Recent studies have suggested that moderately enhanced autophagy in vascular smooth muscle cells during early developmental stage of atherosclerosis may have beneficial effects by maintaining these cells into a more differentiated and contractile phenotype, thereby decreasing cell proliferation and preventing fibrosis 15. In this respect, the signalling role of mild ROS production in promoting dynein-mediated autophagy maturation in CAMs may be protective at the early developmental stage of coronary atherosclerosis secondary to diabetes mellitus. Conversely, excessive ROS production may directly cause lysosomes damage leading to defective autophagy, which results in accumulation of dysfunctional organelles, such as mitochondria, exaggerating the oxidative damages in the vasculature 44,45. Our study may also provide novel insights into understanding the role of autophagy in diabetes. It has been demonstrated that enhanced autophagy compensates the excessive insulin production in β-cells *via* accelerating autophagic degradation of insulin-containing β-granules 46. Therefore, ROS-promoted autophagy maturation may contribute to the maintenance of intracellular insulin content in β-cells in the settings of insulin secretory dysfunction, such as type 2 diabetes.

In conclusion, we demonstrated that in CAMs, NAADP-lysosomal Ca^2+^-regulated dynein activity plays an essential role in ROS-induced autophagy maturation by controlling the trafficking of AP and lysosomes to encounter each other leading to APLs fusion and formation. In addition, ROS/NAADP-Ca^2+^/dynein signalling cascade also contributes to autophagy maturation under high glucose condition implicating a protective role of ROS-sensitive dynein activity during the early stages of hyperglycaemia.
